# Porous Structure of Cylindrical Particle Compacts

**DOI:** 10.3390/mi12121498

**Published:** 2021-11-30

**Authors:** Aidana Boribayeva, Gulfairuz Iniyatova, Aruzhan Uringaliyeva, Boris Golman

**Affiliations:** Department of Chemical and Materials Engineering, School of Engineering and Digital Sciences, Nazarbayev University, Nur-Sultan 010000, Kazakhstan; aidana.boribayeva@nu.edu.kz (A.B.); gulfairuz.iniyatova@nu.edu.kz (G.I.); aruzhan.uringaliyeva@nu.edu.kz (A.U.)

**Keywords:** powder compact, non-spherical particles, microstructure, packing, Voronoi tessellation, porous energy storage and devices

## Abstract

The porous compacts of non-spherical particles are frequently used in energy storage devices and other advanced applications. In the present work, the microstructures of compacts of monodisperse cylindrical particles are investigated. The cylindrical particles with various aspect ratios are generated using superquadrics, and the discrete element method was adopted to simulate the compacts formed under gravity deposition of randomly oriented particles. The Voronoi tessellation is then used to quantify the porous microstructure of compacts. With one exception, the median reduced free volume of Voronoi cells increases, and the median local packing density decreases for compacts composed of cylinders with a high aspect ratio, indicating a loose packing of long cylinders due to their mechanical interlocking during compaction. The obtained data are needed for further optimization of compact porous microstructure to improve the transport properties of compacts of non-spherical particles.

## 1. Introduction

Energy storage technologies have been widely used in the energy supply chain, and further development of these technologies can significantly promote innovations in the energy sector. The bulk particulate materials in the form of particle beds and porous compacts of particles are used in the primary and secondary energy storage processes [1]. For example, fuel pellets from biomass are used as a source of the primary energy as they can be burned to provide the energy when needed [2], and particle-packed beds as a part of the solar thermal systems are used to store the thermal energy in the secondary energy storage processes [3,4]. The combustion process of fuel pellets depends on their size and shape [5], and the morphology of the particle-packed bed determines the heat transfer efficiency of the solar thermal system [6].

Moreover, rising demands for electric vehicles and the widespread application of portable electronics facilitate the development of efficient and environmentally friendly energy storage devices [7,8]. Electrodes in such devices are mainly produced in the form of porous compacts of spherical or, more often, non-spherical particles by tape-casting technology [9]. Our previous investigations confirmed that the electrode performance is governed, among other factors, by the compact microstructure, which in turn depends on the particle properties and process conditions [10,11,12,13,14]. It is known that the shape of particles can significantly affect the particle packing process and, consequently, compact porous microstructure [14,15]. Non-spherically shaped particles are of great interest in current research on powder materials due to their positive impacts on compact mechanical properties, packing density, etc. [16,17]. Thus, there is a need for further analysis of the porous microstructure of non-spherical particle compacts in order to optimize their functional properties for potential applications in energy storage devices.

Various numerical approaches are reported in the literature to model the packing of non-spherical particles, such as the Monte-Carlo method [18], geometrical packing algorithm [19], Discrete Element Method (DEM) [20], and others. The DEM developed by Cundall and Strack [21] is widely applied to simulate the flow of bulk powder consisting of distinct small particles. The multispheres (MS) and superquadrics (SQ) algorithms are most frequently used for generating the non-spherical particles in DEM [22]. A particle of the desired shape is formed as a cluster of overlapping spheres that are glued together using the MS algorithm [23], and the mathematical expressions are used to represent the particle shape as a superquadric by varying the shape parameters in the SQ algorithm [24]. In the present research, the SQ method is applied to generate non-spherical particles due to its cost and time effectiveness.

In recent years, many materials researchers have become interested in studying the packing characteristics of superquadric particles using numerical methods. In particular, many research papers examine the structural and mechanical properties of elliptical particle compacts and compare them with those of spherical particle compacts. For example, Gan et al. [25] conducted research on the packing of ellipses in the form of oblates and prolates to show the change in the contact number of both samples due to their volume differences. The motion of monodisperse elliptical particles in triaxial shear testing was investigated by Zhao et al. [26]. The anisotropy of ellipses orientation was observed during the shearing of packed compact due to the formation of a disordered network of voids. Delaney and Cleary [27] analyzed the effect of particle parameters such as aspect ratio, surface curvature, and blockiness on the packing structure of super-ellipsoids. They confirmed that the rotational degrees of freedom significantly influence the packing characteristics of particles with a high aspect ratio. Additionally, the work of Pereira and Cleary [28] demonstrated the difference in the behavior of packings of cuboids and spheres in a slow rotating cylindrical tumbler. The results of DEM simulations confirmed more pronounced dissipation of energy for cubical particles in comparison with spherical particles during their descending along with the surface layer.

Some recent papers emphasize the applicability of particles of cylindrical shape. As Qian et al. [29] illustrated, the random close packing of monosized equilateral cylinders can be achieved under mechanical vibration by carefully selecting the vibration parameters. Additionally, Zhang et al. [30] used the polydisperse cylindrical particles to generate packing of the fixed bed reactors and simulate the pressure drop and fluid flow in such packings.

The analysis of the morphology of powder compacts has attracted increasing attention from researchers in an effort to improve compact transport properties. Recently, the Voronoi tessellation (VT) approach that refers to the partitioning technique of the void space in a compact has been applied to the analysis of the microstructure of spherical particles compacts [31,32,33,34]. There were also a few attempts to extend the VT approach to compacts of non-spherical particles. For example, Zhao et al. [26] demonstrated the implementation of Voronoi diagram construction for the compact of monodisperse elliptical particles and found the relationship between the surface areas of Voronoi cells and particle shape. Luchnikov et al. [35] also applied the VT concept to study the network of fibers, and Dong et al. [36] derived an extension of the radical VT to compacts of ellipsoidal and cylindrical particles. In the recent works, Zhang et al. [37] claimed that voids generation could significantly influence materials transport and mechanical properties based on the research of three-dimensional VT for granular material with irregular grain shape. Therefore, the VT seems to be a promising method for microstructural analysis of compacts of non-spherically shaped particles.

The aim of this study is to characterize further the microstructure of compacts of cylindrical particles with different aspect ratios. The superquadrics approach is applied to form cylindrical-shaped particles with specified aspect ratios. The DEM is used as a numerical method to simulate samples of cylindrical particles compacts. The quantitative analysis based on Voronoi tessellation is used to analyze the microstructure characteristics of compacts. The obtained data are essential for further analysis of relationships between structural and transport properties of compacts of non-spherical particles. These relationships can be used for the optimization of current and development of new energy storage systems using particulate materials.

## 2. Materials and Methods

### 2.1. Generation of Cylindrical Particles

The particles of cylindrical shape with different aspect ratios are generated using the superquadrics approach. The particle shape and size depend directly on superquadric geometrical parameters such as half-lengths along *x*, *y*, and *z* coordinates and sharpness indices. The superquadric equation for the three-dimensional particles is given as [38]

(1) $$f\left(x,y,z\right)={\left({\left|\frac{x}{a}\right|}^{n_{2}}+{\left|\frac{y}{b}\right|}^{n_{2}}\right)}^{\frac{n_{1}}{n_{2}}}+{\left|\frac{z}{c}\right|}^{n_{1}}-1=0,$$

where *a*, *b*, and *c* are the half-lengths of superquadric along the *x*, *y*, and *z* axes, respectively. The shape sharpness parameter $n_{1}$ defines the shape of cross-sections in the *y*-*z* and *x*-*z* planes, and the parameter $n_{2}$ determines the cross-section shape in the *x*-*y* plane. The geometrical parameters of cylindrical particles are summarized in Table 1. The parameters were chosen so that the generated particles were of the same volume, $V_{p}=21.206\;{\mathrm{mm}}^{3}$.

### 2.2. DEM Simulations

The flow and interaction of particles are simulated using DEM. The particle position and orientation are calculated based on Newton’s second law of motion, taking into account the translational and rotational motions of particles by Equations (2) and (3).

(2) $$m_{i}\frac{d^{2}x_{i}}{dt^{2}}=F_{i},$$

(3) $${\dot{L}}_{i}=T_{i},$$

where $m_{1}$ denotes the *i*th particle mass, $x_{i}$ is the particle position, $L_{i}$ states for the angular momentum of the particle, $L_{i}=I_{i}\cdot \Omega_{i}$, $I_{i}$ is the tensor of inertia, and $\Omega_{i}$ is the angular velocity in the observer-fixed coordinate system.

In the case of non-spherical particles, Equation (3) can be written as [38]

(4) $${\hat{I}}_{i}{\dot{W}}_{i}+W_{i}\times {\hat{I}}_{i}W_{i}=A_{i}^{-1}T_{i}$$

where ${\hat{I}}_{i}$ is the principal tensor of inertia, $W_{i}$ is the angular velocity in the particle-based coordinate system, $W_{i}=A_{i}^{-1}\Omega_{i}$, and $A_{i}$ is the rotation matrix.

The overall sums of the torques, $T_{i}$, and forces, $F_{i}$, acting on an *i*th particle are calculated by Equations (5) and (6) as

(5) $$F_{i}=F_{i,contact}+F_{i,gravity}+F_{i,external},$$

(6) $$T_{i}=T_{i,contact}+T_{i,external}.$$

The rotation matrix *A* is defined by four quaternions $q_{0},\;q_{1},\;q_{2}$, and $q_{3}$ as [39]

(7) $$A=\left(\begin{array}{lll} 1-2\left(q_{2}^{2}+q_{3}^{2}\right) & 2\left(q_{1}q_{2}-q_{0}q_{3}\right) & 2\left(q_{1}q_{3}+q_{0}q_{2}\right)\\ 2\left(q_{1}q_{2}+q_{0}q_{3}\right) & 1-2\left(q_{1}^{2}+q_{3}^{2}\right) & 2\left(q_{2}q_{3}-q_{0}q_{1}\right)\\ 2\left(q_{1}q_{3}+q_{0}q_{2}\right) & 2\left(q_{2}q_{3}+q_{0}q_{1}\right) & 1-2\left(q_{1}^{2}+q_{2}^{2}\right) \end{array}\right)$$

The Hertz-Mindlin-Deresiewicz contact model is used in the present simulations [40]. According to this model, the interparticle contact force is a combination of the normal $F_{n}$ and tangential $F_{t}$ forces. The normal force consists of spring and damping forces and is given as

(8) $$F_{n}=k_{n}\delta_{n}+\gamma_{n}v_{n}$$

Here, $\delta_{n}$ is the normal overlap distance and $v_{n}$ denotes the normal component of the relative velocity.

The normal stiffness $k_{n}$ and normal damping $\gamma_{n}$ coefficients are given as [41]

(9) $$k_{n}=\frac{4}{3}E^{*}\sqrt{R^{*}\delta_{n}},\gamma_{n}=-2\sqrt{\frac{5}{6}}\beta \sqrt{S_{n}m^{*}}$$

where $E^{*}$ is the effective Young’s modulus defined as

(10) $$\frac{1}{E^{*}}=\frac{\left(1-\nu_{1}^{2}\right)}{E_{1}}+\frac{\left(1-\nu_{2}^{2}\right)}{E_{2}}$$

and $\nu_{1},\;\nu_{2},\;E_{1},\;E_{2}$ are the Poisson’s ratios and Young’s modulus of contacted particles, respectively.

The effective mass $m^{*}$ and effective radius $R^{*}$ are given as

(11) $$m^{*}=\frac{m_{1}m_{2}}{m_{1}+m_{2}},R^{*}=\frac{R_{1}R_{2}}{R_{1}+R_{2}}$$

where $m_{1},\;m_{2},\;R_{1},\;R_{2}$ are the masses and radii of contacted particles, respectively. The radius of superquadric particle is calculated as R=1/K, where K is the mean local curvature coefficient, $K=\left(k_{1}+k_{2}\right)/2$, and $k_{1},k_{2}$ are the principal curvature coefficients [22]. The normal overlap distance is defined as $\delta_{n}=X_{1}-X_{2}$. Here, $X_{1}$ and $X_{2}$ are the nearest intersection points between the superquadric surface and the contact line and they are calculated by using an algorithm proposed by Podlozhnyuk et al. [39] and further discussed by Wang et al. [42]. The parameters β and $S_{n}$ are given as

(12) $$\beta =\frac{\ln\left(e\right)}{\sqrt{\ln^{2}\left(e\right)+\pi^{2}}},S_{n}=2E^{*}\sqrt{R^{*}\delta_{n}}$$

where e is the coefficient of restitution.

The tangential force comprises the shear force that accounts for the tangential displacement and the damping force as [43]

(13) $$F_{t}=\min\left[-k_{t}\delta_{t}-\gamma_{t}v_{t},\;\mu F_{n}\right]$$

where $\delta_{t}$ is the tangential overlap distance, $v_{t}$ is the tangential components of the relative velocity, and μ is the coefficient of sliding friction. The tangential stiffness $k_{t}$ and tangential damping coefficients $\gamma_{t}$ are given as

(14) $$k_{t}=8G^{*}\sqrt{R^{*}\delta_{n}},\gamma_{t}=-2\sqrt{\frac{5}{6}}\beta \sqrt{S_{t}m^{*}}$$

Here, $G^{*}$ is the equivalent shear modulus defined as

(15) $$\frac{1}{G^{*}}=\frac{1-\nu_{1}^{2}}{G_{1}}+\frac{1-\nu_{2}^{2}}{G_{2}}$$

where $G_{1}$ and $G_{2}$ are the shear modulus of contacted particles. The tangential stiffness is given as

(16) $$S_{t}=8G^{*}\sqrt{R^{*}\delta_{n}}$$

The packing of cylindrical particles was simulated using Aspherix software [44]. The particle mechanical and physical properties and DEM simulation parameters are summarized in Table 2. The particle and wall material parameters and particle-particle and particle–wall contact parameters were chosen to be close to those used in modeling electrode materials of lithium-ion batteries [45]. However, to decrease the simulation time, Young’s modulus was set to be the same as in the work of [27] and four magnitudes less compared to that of electrode material. Lommen et al. [46,47] confirmed that such calibration of Young’s modulus does not cause significant changes in the packing structure of particle compacts.

The five samples of compacts of monodisperse cylinders with various aspect ratios were simulated by placing 12,000 cylinders at a random position and with random orientation in the domain with a size of 0.07 × 0.07 × 0.15 m and fixed walls. Then, the particles were allowed to settle down under gravity forming the compact, and the simulation continued for a long time until the kinetic energy vanishes to enable particle packing to reach the equilibrium state.

### 2.3. Voronoi Tessellation of Compacts

The Voronoi tessellation of compacts of cylindrical particles was carried out in order to evaluate the compact microstructure. The Voronoi cells were defined in such a way that each cell contains only one cylinder and the void space assigned to this cylinder. The set Voronoi tessellation method developed by Schaller et al. [48] was used to construct Voronoi cells. In this method, the surface of each cylinder is discretized to generate the meshes of points, the Voronoi tessellation is conducted on these meshes for all particles, and, finally, the Voronoi cells belonging to the same particle are merged [26].

The open-source software PySetVoronoi [37] was applied to construct the Voronoi diagram and analyze its properties. The position, shape parameters, and orientation of each particle, such as particle center coordinates, half-lengths along *x*, *y*, *z* directions, and sharpness indices, and quaternions in the global coordinate system, were collected from the output results of DEM simulation. This information was used in PySetVoronoi software to reconstruct cylindrical particles as superquadrics by Equation (1). The spatial orientation of superquadrics was defined by four quaternions.

The volume and surface area of each Voronoi cell in Voronoi diagrams of samples of compacts of monodisperse cylindrical particles S1–S5 were determined using the PySetVoronoi program. Then, the reduced volume of the Voronoi cell was calculated as $V_{c}/V_{p}$, where $V_{c}$ is the volume of the Voronoi cell, $V_{p}$ is the volume of the cylindrical particle, and the reduced free volume of the cell $V_{rc}$ as $V_{rc}=V_{c}/V_{p}-1$ [36]. Similarly, the reduced surface area of the Voronoi cell was estimated as $S_{c}/S_{p}$, where $S_{c}$ is the cell surface area and $S_{p}$ is the cylindrical particle surface area.

## 3. Results and Discussion

### 3.1. DEM Simulation Results

**Figure 1:**
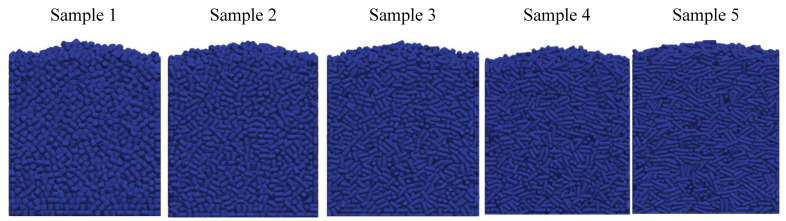
Visualization of compacts of cylinders simulated using DEM.

The visualization of DEM simulations confirmed that the generated cylindrical particles are randomly oriented, their orientation remains random during settling under gravity in the *Z* direction, but they become preferably oriented with their long axes aligned along the *X* and *Y* directions upon contact with particle bed or container bottom. This tendency is more pronounced for cylinders with a larger aspect ratio. The snapshots of cylindrical particle compacts at the end of the DEM simulation are shown in Figure 1. The formation of a larger number of large-size voids is observed in the compact of Cylinder 5 compared to compacts of other cylinders because the mechanical interlocking effect prevents the compaction of high-aspect-ratio cylinders.

### 3.2. Packing Structure Analysis

**Figure 2:**
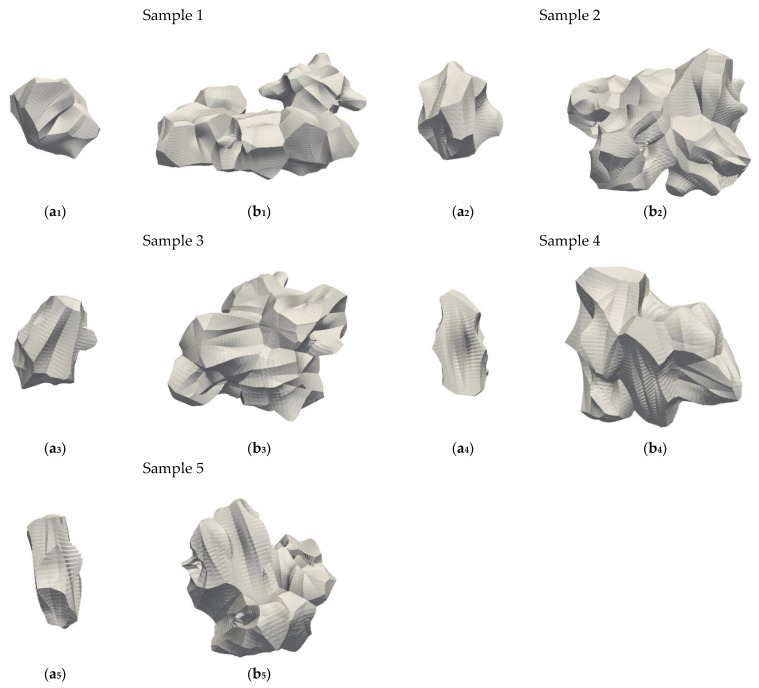
Illustration of Voronoi cells for compacts of cylindrical particles: (**a_1_**–**a_5_**) one cylindrical particle; (**b_1_**–**b_5_**) five cylindrical particles.

The spatial Voronoi tessellation was performed on the simulated samples of cylindrical compacts to divide the compact into the Voronoi cells and allocate void space among particles to the corresponding cells. Figure 2 visualizes Voronoi cells for five samples of compacts of cylindrical particles. In order to better show how void space was discretized, five Voronoi cells were selected. Moreover, a single cell is also shown in the same figure to demonstrate the smoothness of the surface of generated Voronoi cell.

The Voronoi cells of compacts of cylindrical particles have many faces and much more complex geometry than those measured for compacts of spherical particles [33,34]. The Voronoi cells of Samples 4 and 5 are more elongated with fewer faces than those of Samples 1–3 due to the higher aspect ratio of cylindrical particles forming compacts 4 and 5.

**Figure 3:**
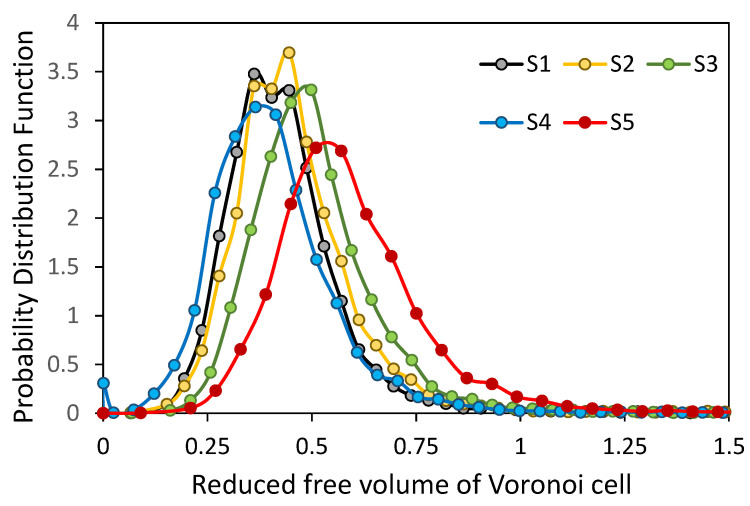
Distribution of reduced free volume of Voronoi cells.

The probability distribution functions of the reduced free volume of Voronoi cells are shown in Figure 3. With an increase in the aspect ratio of cylinders, the maximum value of the probability distribution function decreases, the distribution shifts to the right and becomes broader, except for Sample 4.

**Figure 4:**
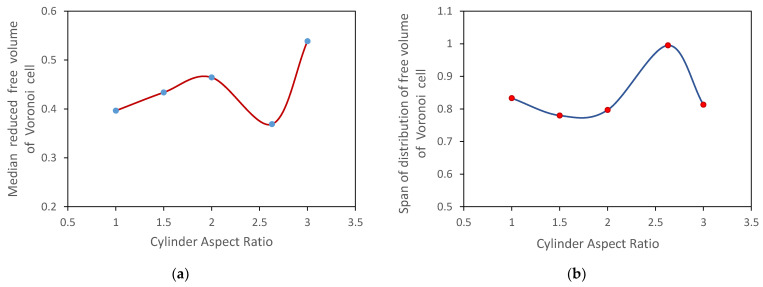
Influence of the shape of cylindrical particles on (**a**) median and (**b**) span of the distribution of the reduced free volume of Voronoi cells.

**Figure 5:**
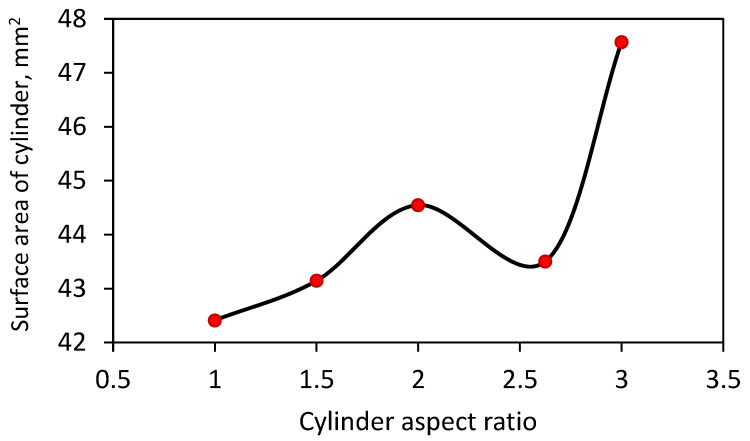
Surface area of cylinders for Samples 1–5.

The medians and the span of the distributions of the reduced free volume of Voronoi cells are shown in Figure 4. With an increase in the cylinder aspect ratio, the median of distribution increases, and the span of distribution changes insignificantly. The distinctive behavior of the probability distribution function of the reduced free volume of Voronoi cells for Sample 4 can be related to the smaller surface area of Cylinder 4 compared to surface areas of Cylinders 3 and 5, as shown in Figure 5. The tendency for the volume-specific surface areas is the same as shown in Figure 5, taking into account that all cylinders are of the same volume.

**Figure 6:**
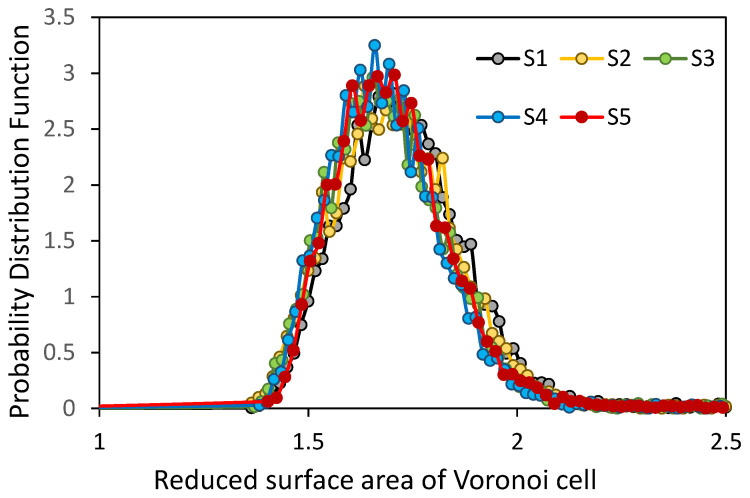
The probability distribution function of the reduced surface area of Voronoi cells.

The probability distribution functions of the reduced surface areas of Voronoi cells are shown in Figure 6. The distributions are broad, and no significant changes in the position and magnitude of the most probable value of the reduced area are observed.

**Figure 7:**
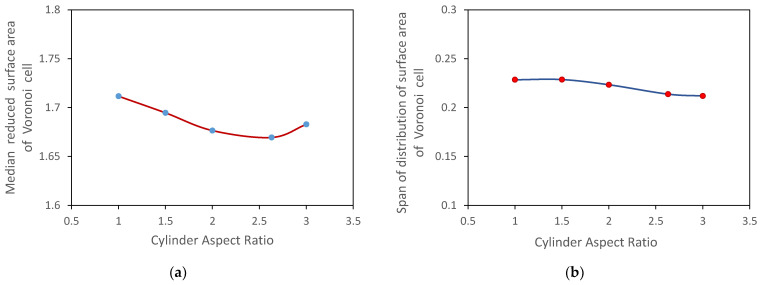
Influence of the shape of cylindrical particles on (**a**) median and (**b**) span of the distribution of reduced surface areas of Voronoi cells.

The medians and the span of the distributions of the reduced surface area of Voronoi cells are shown in Figure 7. With an increase in the cylinder aspect ratio, the median decreases for Samples 1–4 and then increases for Sample 5 composed of cylinders with an aspect ratio of 3. The distribution span decreases slightly for Samples 4 and 5. Dong et al. [36] observed similar patterns for packings of ellipsoids and cylinders.

**Figure 8:**
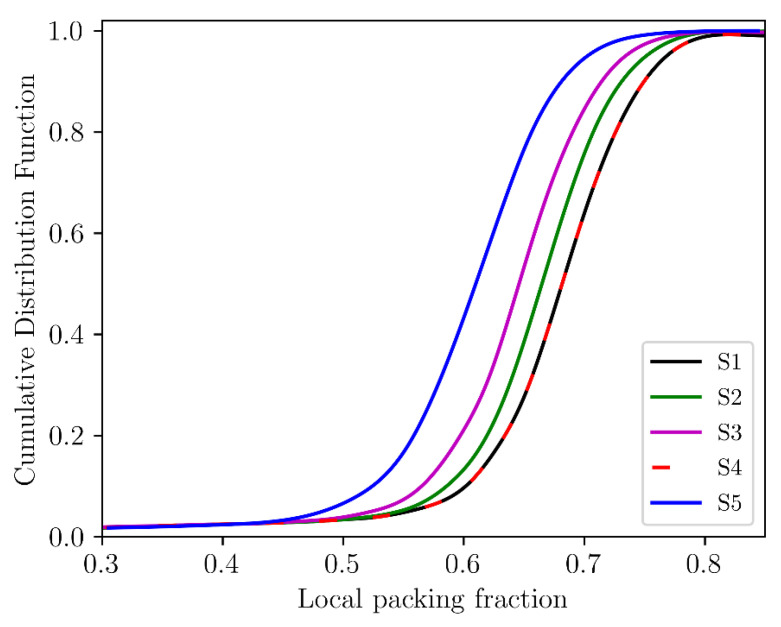
Cumulative distributions of local packing fractions of compacts.

**Figure 9:**
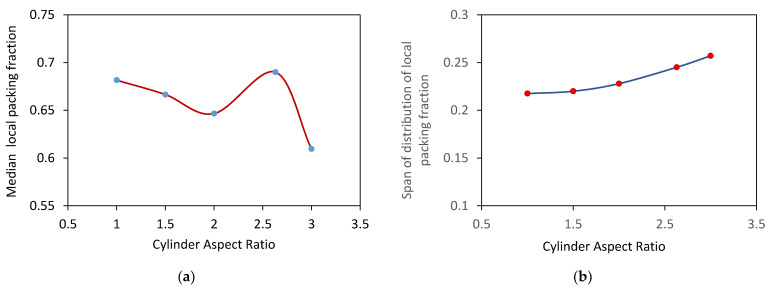
Influence of the shape of cylindrical particles on (**a**) median and (**b**) span of the distribution of local packing fractions.

The cumulative distributions of local packing fractions are illustrated in Figure 8. The local packing fraction was estimated as a reciprocal of the reduced Voronoi cell volume, i.e., $V_{p}/V_{c}$. The median and span of distributions of local packing fractions are shown in Figure 9. The distribution span increases with the aspect ratio of cylindrical particles. Moreover, the distributions shift to smaller packing fractions, and the median packing fraction decreases for compacts of cylinders with high aspect ratios except Sample 4. The compacts of cylinders with high aspect ratios are more loosely packed than compacts composed of cylinders with low aspect ratios due to the possible mechanical interlocking of long cylinders during packing.

The objective of the present paper is to study the morphology of non-spherical particle compacts formed by the free-falling of particles under the action of gravity, as this technique of particle packing is used frequently in the industry. Recently, various techniques have been proposed to produce dense particle packings such as mechanical vibration, snowstorm filling, etc. [49]. The morphology of dense compacts of non-spherical particles generated using these techniques can be the subject of future research.

## 4. Conclusions

The present paper is devoted to the characterization of the porous compacts of non-spherical particles extensively used in energy storage devices. The microstructure of generated compacts of monodisperse cylindrical particles was investigated quantitatively using Voronoi tessellation. The superquadrics were used to represent cylindrical particles with various aspect ratios, and the discrete element method was adopted to simulate the compacts formed under gravity deposition of randomly oriented particles. As a result, it was found that the median reduced free volume of Voronoi cells increases, and the median local packing density decreases for compacts composed of cylinders with a high aspect ratio with the exception of a compact composed of cylinders with relatively low specific surface area. The existence of large voids in the loosely packed compact of long cylinders can be explained by their mechanical interlocking during compaction. Using the obtained data, the optimization of compact porous microstructure could be carried out in the future to improve the transport properties of compacts of non-spherical particles.

## Figures and Tables

**Table 1:** Visualization of cylinders created by using superquadrics approach.

**Samples**					
	Sample 1	Sample 2	Sample 3	Sample 4	Sample 5
	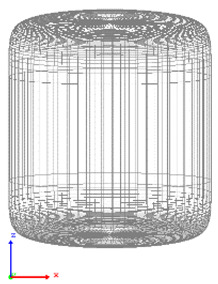	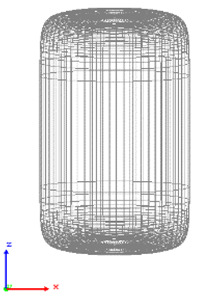	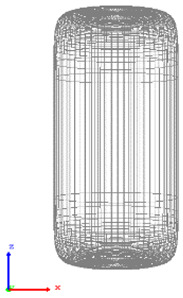	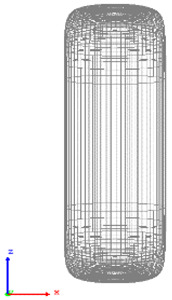	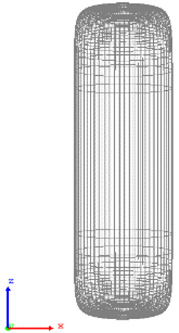
	**Superquadrics parameters**				
a, m	0.0015	0.00131	0.001191	0.0010525	0.00104
b, m	0.0015	0.00131	0.001191	0.0010525	0.00104
c, m	0.0015	0.001966	0.002381	0.002763	0.00312
$n_{1}$	10	10	10	10	10
$n_{2}$	2	2	2	2	2

**Table 2:** Mechanical properties of particles and DEM simulation parameters.

	Properties	Value
Mechanical properties	Young’s modulus, (Pa)	5 × 10^6^
	Poisson ratio	0.4
	Restitution coefficient	0.6
	Friction coefficient	0.4
DEM parameters	Time-step, (s)	1 × 10^−5^
	Gravity, (m/s^2^)	9.81
Particle physical properties	Density, kg/cm^3^	2500
